# Trial participation as avoidance strategy: a qualitative study

**DOI:** 10.1111/hex.12437

**Published:** 2016-01-05

**Authors:** Natalie Armstrong, Elizabeth Shaw, Elaine McColl, Douglas G. Tincello, Paul Hilton

**Affiliations:** ^1^ Department of Health SciencesUniversity of LeicesterLeicesterUK; ^2^ Newcastle Clinical Trials Unit and Institute of Health & SocietyNewcastle UniversityNewcastle upon TyneUK; ^3^ Clinical DeaneryNewcastle UniversityNewcastle upon TyneUK

**Keywords:** avoidance, feasibility studies, interview studies, patient preference, pilot studies, qualitative, randomized controlled trial, stress urinary incontinence, trial participation, urodynamics

## Abstract

**Background:**

Trial participation decisions are often influenced by expectations of potential benefit. Attention has focused on trial participation as a means of securing something seen as desirable, such as experimental treatment. In contrast, we consider a case in which one trial arm involved receiving less than usual care. We explore how this influenced participants’ decisions to participate.

**Methods:**

Semi‐structured interviews with 29 women participating in a pilot trial comparing invasive urodynamic testing (typically normal care) to basic clinical assessment with non‐invasive tests, prior to surgical treatment for stress urinary incontinence. Analysis was based on the constant comparative method.

**Results:**

Invasive tests were something many were aware of and worried about. Participants understood that trial participation meant they might avoid having these tests, and for about one‐third, this was the primary factor motivating participation. A further third mentioned they were not looking forward to tests (if allocated to them) or were lucky to have missed them (if allocated to basic clinical assessment). None of the women appeared to have discussed their desire to avoid having invasive tests with their clinicians.

**Conclusions:**

In contrast to cases in which trial participation is motivated by the wish to secure an intervention not otherwise available, this study reports the opposite – trial participation as an opportunity to avoid having something regarded as undesirable. The option to decline a particular intervention should always be available, and care must be taken to ensure that potential participants are aware that trial participation is not the only possible means of avoidance.

## Background

Decisions to participate in clinical trials are often heavily influenced by expectations of potential benefit, at both a societal and individual level,^1^, ^2^, ^3^, ^4^, ^5^, ^6^, ^7^, ^8^, ^9^ and eligible individuals may be unwilling to be recruited because of the risk of being randomized to receive a placebo.^10^ In terms of individual benefit, most attention to date has been focused on trial participation as a means of securing something that the participant wishes to have, such as access to a new treatment, or a perceived better standard of care. As an example, McCann *et al*.^7^ interviewing participants in a trial comparing medical and surgical interventions for gastro‐oesophageal reflux disease, while emphasizing that willingness to contribute to the collective good was a key motivating factor, also found ‘conditional altruism’ evidenced by a feeling that trial participation might bring: an opportunity for closer specialist review; (faster) access to surgery; and more careful monitoring of disease progress.

Motivations based on potentially unrealistic or misinterpreted expectations of individual benefit may be problematic,^11^ particularly given that evidence shows that although trials are only conducted when there are reasonable grounds to expect benefit from the trialled interventions (and no evidence of harm), new treatments are found to be better than existing ones only just over half the time, and new treatments may indeed perform less well than existing ones.^12^ In addition, there seems only weak evidence to suggest that participation in clinical trials *per se* has a positive effect on individual participants’ outcomes and that the effect seems to be larger in trials where an effective treatment already exists and is included in the trial protocol.^13^, ^14^

While the current literature on this topic consists of cases in which motivation to participate is driven, at least in part, by a desire to secure access to something that the participant wishes to have, this study looks at a new and interesting twist in which one trial arm comprised receiving something less than usual care. We explore how this influenced participants’ decisions to take part in the trial.

The INVESTIGATE‐I study was a mixed methods feasibility study including a pragmatic multicentre ‘rehearsal’ pilot randomized controlled trial (RCT) of invasive urodynamic testing compared to basic clinical assessment before surgical treatment for stress urinary incontinence in women; it was funded under the UK National Institute for Health Research Health Technology Assessment programme (NIHR‐HTA) programme.^15^, ^16^ Urinary incontinence, while rarely life‐threatening, may seriously influence the physical, psychological and social well‐being of affected individuals. Prevalence figures range from 5 to 69% in women 15 years and older, with most studies reporting prevalence in the range 25–45%.^17^ More severe urinary incontinence is reported in 4–7% of women under the age of 65, and around five million women over 20 years of age may be affected in England and Wales.^18^

Several methods are used in the assessment of urinary incontinence in order to evaluate function of the lower urinary tract and guide decisions about the most appropriate way to manage the condition. These include non‐invasive tests (such as free urine flow rate and post‐void residual volume), but some tests require catheterization (such as conventional cystometry or videourodynamics) and are therefore regarded as invasive. Such invasive tests involve having the bladder filled, provocation tests to show leakage and then voiding, all in the presence of healthcare professionals.

Despite its widespread clinical use over four decades, the appropriate position of invasive urodynamic testing in the diagnostic pathway remains unclear. The UK National Institute for Health and Care Excellence (NICE),^19^ the NIHR‐HTA programme,^20^ the Cochrane Collaboration^21^ and the International Consultations on Incontinence^22^ have all undertaken systematic reviews on the subject and all emphasize the lack of high‐quality primary research confirming clinical utility.

While serious morbidity associated with invasive urodynamic testing is rare, up to 20% of women with sterile urine prior to investigation may develop bacteriological evidence of urinary tract infection subsequently.^23^, ^24^, ^25^, ^26^ Anxiety and embarrassment on the part of those undergoing invasive urodynamic testing are commonly reported,^27^ although not all women will experience high anxiety levels.^28^ Over a quarter of women experience pain during investigation.^29^

In England, the current guidance from NICE suggests that invasive urodynamic testing is not required prior to conservative treatments and that, while it may be needed in more complex clinical scenarios, there is no evidence to support its use prior to surgery where the diagnosis of stress urinary incontinence is likely based on clinical assessment alone.^30^ Within the INVESTIGATE‐I study protocol, invasive urodynamic testing was described as the intervention arm, and clinical assessment with non‐invasive tests, but without invasive testing, as the control arm. It should be noted, however, that the use of invasive urodynamic testing for these patients is currently commonplace in the UK, and indeed could be more accurately described as ‘usual care’.^31^, ^32^, ^33^ We recognize that this is something of an anomaly, but felt at the time of protocol development that the more active investigation strategy should be designated as the intervention and the less active as the control.

As part of our pilot work for a possible future RCT assessing the value of invasive urodynamic testing in patients with urinary incontinence, we sought to explore women's views on the acceptability of randomization either to invasive testing or to clinical assessment alone (without invasive testing).

## Methods

We conducted semi‐structured interviews with women who had participated in a pilot trial comparing invasive urodynamic testing to basic clinical assessment with non‐invasive tests prior to surgical treatment for urinary incontinence.^15^ While we also invited all women who did not agree to join the pilot trial (59) as soon as possible thereafter for interview, none agreed to do so.

Interviews were carried out to explore women's understandings and experiences of the study (including the initial approach, baseline data collection and 6‐month follow‐up), the consent process and their decision to participate (including how they had made the decision, how easy or difficult they had found this, who they had discussed it with), and any perceived barriers to or facilitators of participation in a future definitive RCT. Purposive sampling was used to include women from a range of ages, trial participation status (randomized and retained to final follow‐up; randomized but did not provide full follow‐up data), allocation status (invasive urodynamic testing or basic assessment), treatment received (surgery or conservative management) and study site.

Women were approached at the end of the trial, so as to capture both their reasons for agreeing to participate and their overall experience of taking part in the study (interviews typically took place 10 months after the decision to participate was made). A specific participant information leaflet was provided for the interview study, and written consent was obtained from all interviewees. The interviews were carried out face to face by an expert qualitative interviewer (see Acknowledgements) and were audio‐recorded and transcribed verbatim.

The interviews were semi‐structured, using a prompt guide with broad topic areas, but the emphasis was on encouraging women to discuss their own perspectives freely and allowing them to raise issues that were important to them. The interviewer prompted as appropriate to ensure that all views were fully explained, and the meaning of participants’ responses clear. The prompt guide was developed from a literature review and discussions within the project team and was modified as the interviews progressed to incorporate issues raised by earlier interviewees.

Data collection continued until saturation of themes was reached and interviews no longer generated new concepts. Analysis was completed by ES and NA based on the constant comparative method,^34^ and aided by NVivo 10 (QSR International, Warrington, UK) qualitative software. Transcripts were read three to four times and open codes initially applied line by line to the data to represent the meaning or significance of each sentence or group of sentences. Generation of the open codes proceeded sequentially, with no attempt at this stage to impose any framework on the data. The open codes were then incrementally grouped into organizing categories or themes. These categories were modified and checked constantly as further open codes were incorporated as analysis proceeded. When categories had been created to express all of the open codes, explicit specifications were written for each of the categories to assist in determining under what circumstances data should be assigned to any given category. The categories and their specifications (the coding scheme) were then programmed into NVivo 10 software. The coding scheme was then used to process the data set systematically by assigning each section of text to a category, according to the category specifications.

For research governance purposes, the INVESTIGATE‐I study as a whole (including these patient interviews) was reviewed by Newcastle & North Tyneside 1 Research Ethics Committee and given a favourable opinion (Ref 10/H0906/76). NHS R&D approval was provided by each participating trial centre.

## Results

### Details of the sample

A total of 111 pilot trial participants were initially invited to take part in an interview; 36 women indicated they were willing to be interviewed. Of these, 29 were interviewed, two withdrew from the interview study before the interview could be arranged, one had moved and so was no longer covered by our research governance approvals, and four were not interviewed as they were from groups already well represented in the sample. Interviewees were between 35 and 75 years of age, came from five of the seven trial centres and included participants from both trial arms (16 received invasive testing; 13 received basic clinical assessment). Twenty‐seven women subsequently had surgery, one received non‐surgical treatment, and one declined surgery.

### Awareness of invasive urodynamic testing, and a wish to avoid it

The specific nature of the trial and the intervention being assessed was an important factor for many women when making decisions about participation. The possibility of having invasive urodynamic testing (which tends to constitute usual care in the UK) performed prior to any surgical treatment was something that many were already aware of, and worried about, prior to being invited to participate in the trial. This awareness had typically come either through their own prior personal experience or through hearing about invasive urodynamic testing from friends and family members who had undergone the test themselves. Concerns typically centred on issues around embarrassment and pain or discomfort.

I had spoken to other people who had had the same operation as I was going to have and they had told me that the worst part about the operation, apart from being in hospital and having the operation and the discomfort afterwards, was having the tests beforehand and they said it just felt like there was a lot of discomfort and, you know, it's just not a very nice experience.

(Interviewee 08, basic clinical assessment)

Well the diagnostic is a bit embarrassing I think, erm […] it's embarrassing enough to have a, a sort of situation like this [incontinence] but when you have got to go and confront people with it as well.

(Interviewee 14, basic clinical assessment)

### Avoidance as a primary motivator for participation

The possibility of avoiding having invasive urodynamic tests was discussed as the primary motivation to participate in the trial by about one‐third of the women we interviewed:

There was a 50:50 chance I wouldn't have to have urodynamics, which I really didn't want to have.

(Interviewee 01, urodynamics)

What really worried me was having all the bladder tests beforehand because I felt quite stressed about things like that and I was told there was a chance if I entered the trial I might still have to have them but there was a chance I might not have to have them, which was quite a good incentive.

(Interviewee 05, basic clinical assessment)

While typically discussed as wishing to avoid a form of testing that they would find unpleasant in its own right, some women also talked about wishing to avoid invasive testing as a means of securing quicker access to surgery for their continence problems:

CAN YOU REMEMBER WHAT YOUR FIRST REACTION WAS WHEN YOU WERE TOLD ABOUT THE TRIAL?

Thought great…Because I had heard that it was quite long winded and a slow process through various different tests and there was one of the tests that I had heard was quite horrible as well I can't remember what it's called now, but there was, and sometimes it sort of just took sort of 6 or 9 months to even get that far, you know and then it was still sort of on‐going so when they said, you know we are doing this trial because we are not sure whether that's actually necessary and if you are chosen for the trial you bypass all that I just thought great (laughs) because obviously it's not something you want to keep having a problem with, you want to get it sorted as quickly as possible don't you?

(Interviewee 10, basic clinical assessment)

### Reactions to trial allocation

In addition to those who discussed avoidance of invasive testing as the primary motivation for their trial participation, a further third of those interviewed discussed invasive testing in negative terms when talking about their trial allocation. Those randomly allocated to receive invasive testing stressed how they were not looking forward to the tests and hoped they might have been able to avoid them:

We were laughing and I said oh I am bound to be picked to do it [IUT] because (laughs) it's one of them things you think, God I hope I am not picked to do it and then they go oh yes you have been picked to do it.

(Interviewee 07, urodynamics)

She asked a few questions, you know, am I sure I want to join the study and things? And I said yeah, it seems absolutely fine, I said anything not to have, see if I get a 50/50 chance not to have the test, you know!

WERE YOU NOT LOOKING FORWARD TO THOSE TESTS THEN?

No, I wasn't!

YOU WERE ALLOCATED URODYNAMICS THOUGH, WEREN'T YOU?

Yes, unfortunately, but never mind.

(Interviewee 31, urodynamics)

Those allocated to the trial arm not receiving invasive tests typically reported being pleased with this outcome, and felt they were lucky to have missed them:

And I was probably [one of] the lucky ones because I didn't have to the test (laughs)…And I have heard that the test, that it can be quite embarrassing.

(Interviewee 04, basic clinical assessment)

The trials nurse rang me and informed me that I had actually managed to avoid the invasive research.

AND HOW DID YOU FEEL ABOUT THAT?

Elated! I really was quite elated because it did sound quite uncomfortable but as I said, I would have gone through with it, had I had to.

(Interviewee 28, basic clinical assessment)

### The potential to avoid invasive testing outside of the trial context

It is interesting given the strong preference expressed by some women to avoid invasive testing that none mentioned the possibility of doing so outside of the trial context. Certainly, none of the women we interviewed appeared to have discussed their desire to avoid having invasive urodynamic testing with their clinicians prior to receiving the invitation to participate.

One participant who discussed the possibility of avoiding invasive testing as the primary motivator for her trial participation had significant concerns about taking part in the trial. She talked about her participation in a previous research study when pregnant, and appeared to have found this very traumatic. However, her wish to avoid invasive testing was sufficiently strong to overcome this and, despite having promised herself she would never participate in research again, she did agree to join INVESTIGATE‐I in order to do so:

WHAT WAS YOUR FIRST REACTION WHEN YOU WERE TOLD ABOUT THE TRIAL FIRST OF ALL?

Probably to not take part, because I promised myself I'd never take part in any trials. I'd previously been involved in a trial when I was pregnant with my first child and it didn't affect the delivery but we were at a different hospital than we would've chosen had we not been part of that trial and I had a very traumatic delivery which caused the incontinence, and it was quite…caused quite a lot of damage, well my child had brain injuries as a result, and so I was quite…I promised myself I'd never get involved in a trial again […] but the big carrot was potentially not having the bladder function tests.

SO DID YOU THINK THEN THAT YOU, IF YOU'D GONE DOWN A ROUTE OF NOT BEING PART OF THE STUDY THAT YOU WOULD HAVE DEFINITELY HAD TO HAVE THE BLADDER FUNCTION TESTS?

Yes, I would have done.

(Interviewee 05, basic clinical assessment)

## Discussion

Being asked to participate in a clinical trial comparing invasive urodynamic testing to basic clinical assessment with non‐invasive tests surfaced a previously undeclared preference for avoiding the invasive urodynamic tests that tend to comprise usual care. Therefore, while the current literature on personal benefit as a motivation for trial participation is often focused on the wish to secure something, this paper has demonstrated that in some cases trial participation can be motivated by a wish to avoid having something that the potential participant regards as undesirable. Of course, one might equally describe this as a desire to secure a non‐invasive investigation strategy. However, as we have shown in the introduction, despite NICE guidance to the contrary, invasive urodynamic testing is in essence current ‘usual care’ in the UK.^31^, ^32^, ^33^ It was also noted that, while several women expressed a wish to avoid invasive testing, or relief that they had performed so, none expressed a positive wish to undergo a non‐invasive investigation strategy. We opine therefore that avoidance is the issue here.

Recognizing the potential for trial participation to be a means of avoidance highlights some important issues. None of the women we interviewed appeared to have discussed their desire to avoid having invasive urodynamic testing with their clinicians prior to receiving the invitation to participate. This is mirrored by the clinician‐focused elements of the INVESTIGATE‐I pilot study in which there was often a clear preference on the part of clinicians for use of invasive urodynamic testing (not always for reasons of pure clinical utility) and importantly no mention of involving patients themselves in decisions about whether or not to do so in any particular clinical case.^32^, ^33^ It is an interesting paradox that agreeing to randomization, which is so often seen as a ceding of control over decision making, in this case was perceived by women as a potential way of gaining a degree of control over what happened to them.

That many patients seemingly had not thought it possible to discuss their preference to avoid invasive urodynamic testing with their clinicians outside of the trial context is perhaps not surprising as it may be difficult for patients to find information and act to try to secure their preferences, not least because many women having invasive urodynamic testing report not feeling fully informed about it or fully understanding it beforehand.^35^ In many cases, it would seem unlikely that invasive urodynamic testing is ‘what women want’.^36^ This undisclosed preference to avoid invasive tests, coupled with NICE's current position that there is no evidence to support the use of these prior to surgery where the diagnosis of stress urinary incontinence is likely based on clinical assessment alone, would seem to suggest a need to move towards use of less invasive tests in these simpler cases, and to more fully involve patients in making decisions about possible use of invasive tests in more complex cases. This latter would mean presenting all options explicitly and in an unbiased way in order to enable patients to make an informed decision – something that may require clinicians to significantly change their approach.

In relation to trials, patient preferences can be understood as evaluations of an intervention in terms of its desirability both in relation to expectations about the process and outcome of any intervention and the perceived value of these. Taking account of patient preferences within trials is not simple, and the effects these can have on any trial's conduct and the validity of the resulting data are complex.^37^ For example, a separate urinary incontinence trial led by PH compared colposuspension with tension‐free vaginal tape.^38^ The issue in this case was that there was differential drop out prior to surgery, with more participants dropping out of the more invasive colposuspension arm. The exploration of the biases this may have caused was complex. In the case of INVESTIGATE‐I, it is worth noting that of 11 participants withdrawing from the study, 5 did so within 6 weeks of randomization; one was randomized in error, but the other four had been randomized to receive invasive urodynamic testing.^15^

While some interventions may only be available within the context of a trial (such as experimental tests or treatments), the option to decline a particular intervention (whether therapeutic or diagnostic) should always be available to patients and care must be taken to ensure that potential participants are aware that trial participation is not the only possible means of avoidance. While there has been a significant focus in recent years on the need to move towards shared decision making between patients and clinicians, this is not yet the norm within health care and is perhaps most usually associated with treatment interventions rather than the investigations and tests that may be involved within the preceding diagnostic process.

This study has some limitations. It appears that the possibility of avoiding invasive testing was a motivating factor in deciding to join the trial for a proportion of women, in the knowledge that this gave them a 50% chance of avoiding investigation. We also invited for interview all women (59) who, while eligible and invited, had declined to participate in the INVESTIGATE‐I pilot trial; none were willing to take part. We therefore do not know to what extent preferences around invasive testing may have been a factor in their decision making. Additionally, we opted to invite participants for interview at the end of the trial, so as to capture both their reasons for agreeing to participate and their overall experience of taking part in the study. This inevitably meant that interviews took place sometime (typically about 10 months) after the decision about whether or not to participate in the trial had been taken. Issues of recall bias and/or *post hoc* rationalization cannot therefore be ruled out.

Notwithstanding these limitations, this study does suggest some important areas for consideration and future research. Perhaps the most important of these is the need to ensure that potential participants are aware that trial participation is not the only possible means of avoidance, although how this can best be achieved in practice will require further work. The official guidance in the UK on providing information and gaining informed consent for research suggests that interactive questioning of potential participants within the consent process can aid understanding and may highlight areas that potential participants have misunderstood.^39^ It may be that patients are not aware of the possibility to decline any particular intervention, particularly if this relates to diagnostic testing rather than treatment, and so understand trial participation to be the only possible means of avoidance. Those taking informed consent in these contexts should be alert to this possibility and be prepared to advise patients accordingly.

## Funding

This project was funded by the NIHR‐HTA programme (reference 09/22/136). The views and opinions expressed herein are those of the authors and do not necessarily reflect those of the Department of Health. The authors also acknowledge the support of the NIHR, through the Comprehensive Clinical Research Network.
